# Heme utilization by the enterococci

**DOI:** 10.1093/femsmc/xtae019

**Published:** 2024-07-02

**Authors:** Debra N Brunson, José A Lemos

**Affiliations:** Department of Oral Biology, University of Florida College of Dentistry, 1395 Center Drive, Gainesville, FL 32610, United States; Department of Oral Biology, University of Florida College of Dentistry, 1395 Center Drive, Gainesville, FL 32610, United States

**Keywords:** enterococci, home, pathophysiology, catalase, cytochromes, biofilm

## Abstract

Heme consists of a tetrapyrrole ring ligating an iron ion and has important roles in biological systems. While well-known as the oxygen-binding molecule within hemoglobin of mammals, heme is also cofactor for several enzymes and a major iron source for bacteria within the host. The enterococci are a diverse group of Gram-positive bacteria that exist primarily within the gastrointestinal tract of animals. However, some species within this genus can transform into formidable opportunistic pathogens, largely owing to their extraordinary adaptability to hostile environments. Although enterococci cannot synthesize heme nor depend on heme to grow, several species within the genus encode proteins that utilize heme as a cofactor, which appears to increase their fitness and ability to thrive in challenging environments. This includes more efficient energy generation via aerobic respiration and protection from reactive oxygen species. Here, we review the significance of heme to enterococci, primarily the major human pathogen *Enterococcus faecalis*, use bioinformatics to assess the prevalence of hemoproteins throughout the genus, and highlight recent studies that underscore the central role of the heme–*E. faecalis* relationship in host–pathogen dynamics and interspecies bacterial interactions.

## Teaser

The significance of heme to Enterococcus faecalis is reviewed while also identifying the prevalence of hemoproteins throughout the enterococci and highlighting gaps in knowledge in enterococcal mechanisms of heme homeostasis.

## Introduction

Heme is an important nutrient cofactor for certain aerobe and anaerobe bacteria as it serves important roles such as energy generation, sensing and defense against reactive oxygen species (ROS), sensing of nitric oxide, as a trigger for enhanced biofilm formation, and can often be used as an iron source during host infection (Winstedt et al. 2000, Frankenberg et al. 2002, Pedersen et al. 2012, Choby and Skaar 2016, Layer 2021, Lee-Lopez and Yukl 2022). In mammals, heme is primarily found as the cofactor to hemoglobin within red blood cells, but it is also found in abundance as a cofactor in myoglobin within muscle cells and many other proteins (Ponka 1999). Additionally, levels of heme within the gut of mammals and other animals vary widely depending on the diet (Khalili et al. 2017). The varied bioavailability and distribution of heme within the host makes mechanisms for heme acquisition or synthesis, utilization, and even detoxification important fitness factors for some species of commensal, opportunistic, or pathogenic bacteria (Gruss et al. 2012, Baureder and Hederstedt 2013). Several reviews have highlighted the role of heme in bacterial physiology to include the importance of hemophores to the evolution of the oral and gut microbiota (Olczak et al. 2024), the heme synthesis pathways (Yang et al. 2024), and heme acquisition and tolerance mechanisms in Gram-positive bacteria (Wang et al. 2023).

**Figure 1.**
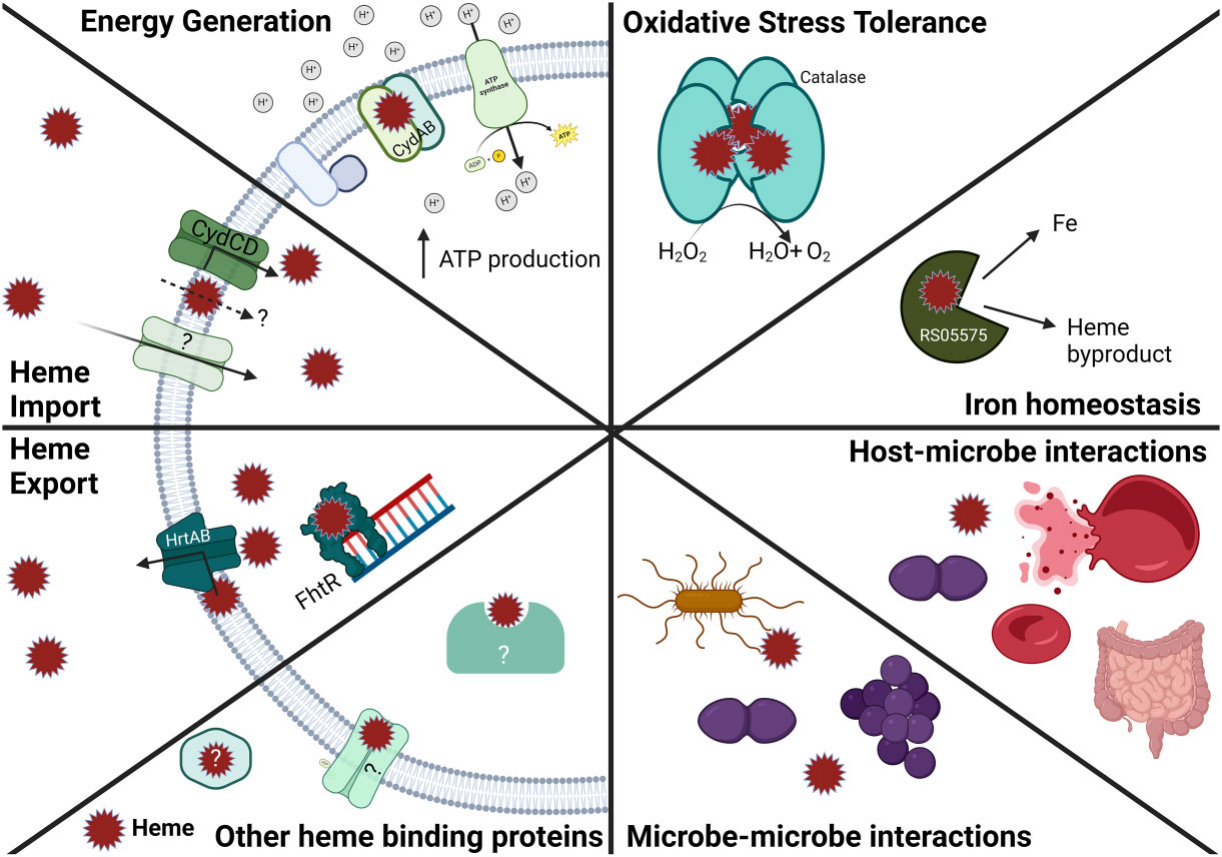
Established and predicted mechanisms of heme acquisition, utilization, and export in enterococci. Heme is an essential cofactor of cytochrome *bd* and catalase and can be an important source of iron within the host. Toxicity is managed by a heme-sensing regulator FhtR and an export system HrtAB, but import is likely mediated by CydCD and other unidentified import systems(s) or through passive diffusion across the membrane. Heme has also been established as an important molecule in microbe–microbe and host–microbe interactions in which hemoproteins of host and bacterial origin are degraded and the freed heme is used by *E. faecalis* to enhance biofilm formation. Created using BioRender.

The enterococci are gut dwelling Gram-positive diplococci that arose 425–500 million years ago and have been coevolving in the guts of animals for millions of years (Lebreton et al. 2014, 2017). Currently, the *Enterococcus* genus comprises a little over 60 species and representatives are found in the guts of virtually all animals including insects, aquatic animals, reptiles, amphibians, birds, and mammals (Lebreton et al. 2014, 2017). In addition to being gut commensals, *Enterococcus faecalis* and *Enterococcus faecium*, the two major human colonizers, are also opportunistic pathogens that survive and tolerate multiple stressors commonly found within the host and hospital environments (Lebreton et al. 2014, 2017, Schwartzman et al. 2023). These stressors often include low nutrient availability in the environment, host-imposed starvation, fierce competition with other microbes for available nutrients, fluctuations in environmental pH and osmolarity, disinfectants such as chlorhexidine, and production of ROS or antimicrobial peptides by the host or competing microbes (Gaca and Lemos 2019, García-Solache and Rice 2019, Gaston et al. 2020). While the enterococci do not require heme for growth nor can they synthesize their own heme, they do possess heme-dependent enzymes that can aide the cell in overcoming many of these stressors including more efficient energy generation, protection against ROS, and utilization of heme as an iron source (Fig. 1) (Winstedt et al. 2000, Frankenberg et al. 2002, Brunson et al. 2023).

Most of what is known concerning heme utilization in enterococci comes from studies performed with *E. faecalis* and include identification of heme-dependent catalase and cytochromes, heme-binding transmembrane proteins, and characterization of a heme-sensing regulator and heme export system (Winstedt et al. 2000, Frankenberg et al. 2002, Baureder et al. 2012). Additionally, a heme-binding protein annotated as either a coproporphyrinogen III oxidase or a heme chaperone has been identified but not characterized (Wilkinson et al. 2023). This review will primarily focus on the significance of hemoproteins to the physiology and virulence of *E. faecalis*. Considering that very little is known about heme utilization by other enterococci, we also used bioinformatics to assess the presence/prevalence of each *E. faecalis* hemoprotein to potential homologues in other enterococci.

## Energy generation by cytochrome *bd*

Enterococci primarily derive energy from fermentation of sugars with only a few species having an additional capacity to conserve energy via aerobic respiration (Ramsey et al. 2014). One such species, *E. faecalis*, can actively respire, but only in the presence of exogenous heme that is the cofactor of the respiratory enzyme complex cytochrome *bd*. The *E. faecalis* cytochrome *bd* consists of two polypeptides, CydA and CydB. The CydA protein contains the three distinct heme-binding domains, cytochrome *b_558_*, which is the site of quinol oxidation, as well as *b_595_*and *d* (Borisov et al. 2021). The oxidase activity of the enzyme generates a transmembrane gradient that ultimately drives ATPase activity. The second polypeptide is encoded by *cydB*, and while it does not contain heme, it is believed to be paralogous to *cydA* having arisen from a duplication event (Cook and Poole 2016). Together, CydA and CydB form a heterodimeric complex that is stabilized by interactions of alpha helices α3, α4, and α9 (Borisov et al. 2021). Importantly, supplementation with heme to the growth media has been shown to enhance both planktonic growth (Painter et al. 2017) and biofilm formation in *E. faecalis* (Ch’ng et al. 2022).

**Figure 2.**
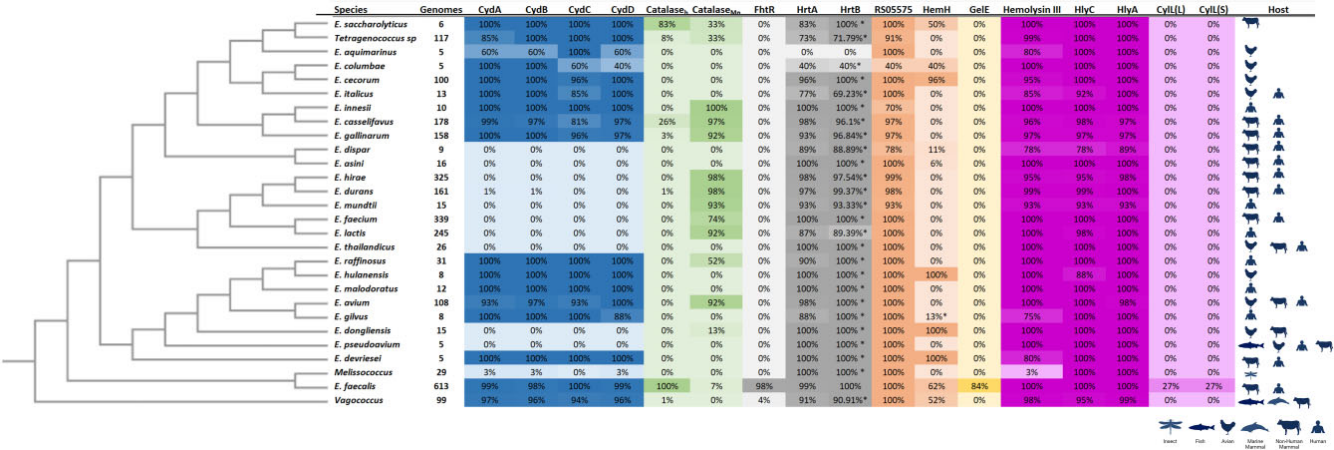
Phylogenetic relationships of the enterococci and prevalence of proteins involved in heme utilization. The BV-BRC database was searched for enterococcal species with at least five sequenced genomes of good quality. Representative genomes were chosen for phylogenetic tree construction using the BV-BRC bacterial genome tree tool. The total number of genomes used to search for potential hemoprotein homologues are shown. A genome was considered positive for a particular hemoprotein if the query and subject coverage were >90% and the amino acid identity was >50%. The percentage of species that was found to be positive for each protein is indicated numerically and by heat-map, where darker colors indicate proteins are more prevalent within isolates of the species. * Indicates the presence of a potential homologue with 40%–50% amino acid identity. The phylogenetic tree was constructed using the BV-BRC bacterial genome tree tool. A representative genome from each species was chosen and 1000 genes were used in the Codon Tree method in which both amino acid and DNA sequences from single copy genes were analyzed using RAxML. The most common host(s) each species was isolated from is shown. Created using BioRender.

The Bacteria and Viral Bioinformatics Resource Center (BV-BRC) combines several tools and databases for bacterial bioinformatic analyses (Olson et al. 2023). As of March of 2024, this database contained whole genome sequences of 66 enterococci isolated from environmental, animal, or human sources. Throughout this article, we utilized this database to search for homologues of *E. faecalis* hemoproteins in other members of the genus to gain a broader insight into the significance of heme to other enterococci. For these analyses, we only included enterococcal species in which there were at least five genomes with good quality sequencing, which gave us 25 species of enterococci to search for hemoproteins. The *E. faecalis* OG1RF genome was used as the basis for most of our comparisons because it is the most used strain for *E. faecalis* genetic manipulations and generally accepted as a good representative of the species (Bourgogne et al. 2008, Dale et al. 2018). In addition, we included the closely related (and former enterococci) *Vagococcus, Melissococcus*, and *Tetragenococcus* species (Lebreton et al. 2014, 2017, Schwartzman et al. 2023). A positive hit was considered if the protein identified shared greater than 50% amino acid identity and greater than 90% query and subject coverage. First, we probed the prevalence of CydA (OG1RF_RS08540) and CydB (OG1RF_RS08535) across strains of *E. faecalis* and other species of enterococci. We found that nearly all strains of *E. faecalis* (98%–99%) encode CydA and CydB while both genes are completely absent in *E. faecium* strains. Overall, the distribution of cytochrome *bd* in the genus is uneven with several species (e.g. *E. avium, E. casseliflavus, E. cecorum*, and *E. gallinarum*) encoding CydA and CydB homologues while other species including *E. durans, E. hirae*, and *E. lactis* do not. Additionally, we found a high prevalence of CydA and CydB in Tetragenococci and Vagococci, while both proteins can be found rarely in Melissococci (Fig. 2).

## The transmembrane proteins CydC and CydD are associated with cytochrome *bd* assembly and heme uptake

The *cydA* and *cydB* genes are part of an operon, *cydABCD*, in which *cydC* and *cydD* encode integral membrane proteins each with an ATPase domain. In *Escherichia coli*, the CydCD complex is required to export thiol-containing compounds, uses heme as a cofactor to support its ATPase activity, and is required for cytochrome *bd* assembly (Pittman et al. 2002, Yamashita et al. 2014, Holyoake et al. 2015, 2016, Shepherd 2015, Poole et al. 2019). Recently, it has also been demonstrated that the CydCD complex of *E. coli* captures heme from the cytoplasmic membrane and uses a trap-and-flip mechanism to transport heme to the periplasmic space (Wu et al. 2023). In *E. faecalis*, CydCD was also shown to be required for cytochrome *bd* assembly (Baureder and Hederstedt 2012) and was linked to heme transport as intracellular heme was below the detection limits in whole cell lysates of transposon mutants disrupted in either *cydC* or *cydD* (Ch’ng et al. 2022). Accordingly, the disruption of *cydC* or *cydD* also led to a defect in biofilm biomass when grown in the presence of heme (Ch’ng et al. 2022). Taken together, these data suggest that CydCD is a major driver of heme uptake in *E. faecalis* and essential for activation of the cytochromes. However, a few points remain unclear. First, in contrast to *E. coli* CydCD, which was shown to transport heme from the cytoplasmic membrane to the periplasmic space, *E. faecalis* CydCD appears to play a role in heme import. While it lacks validation, it is tempting to speculate that extracellular heme becomes associated with *E. faecalis* membranes and then is transported to the cytoplasm by a similar trap-and-flip mechanism. Second, strains lacking CydCD retain catalase activity, an enzyme whose function is reliant upon the presence of intracellular heme (Baureder and Hederstedt 2012). Retention of catalase activity indicates the presence of alternate heme import mechanisms that have yet to be identified. Notably, enterococcal genomes do not encode homologues of well-characterized heme scavenging and import systems found in related bacteria such as *Streptococcus pyogenes* or *Staphylococcus aureus*, making the identification of heme importers in the enterococci a difficult endeavor (Cassat and Skaar 2012, Ouattara et al. 2013, Pishchany et al. 2014, Zhang et al. 2017, Chatterjee et al. 2020, Wang et al. 2023). Considering the relevance of heme to bacterial pathophysiology, the identification of the heme importers of *E. faecalis* should be a priority in the field. Like cytochrome *bd*, both CydC (OG1RF_RS08525) and CydD (OG1RF_RS08530) were found in almost all *E. faecalis* strains, but not in *E. faecium*, and most species that possess CydA and CydB also had a prevalence for CydC and CydD (Fig. 2).

## Protection against oxidative stress by catalase

Except for *E. faecalis*, the enterococci are generally classified as catalase negative (Baureder and Hederstedt 2013). Previous studies have demonstrated that heme is an essential cofactor for catalase in *E. faecalis* and that catalase protects against exogenous H_2_O_2_ but not when H_2_O_2_ is produced endogenously as a byproduct of glycerol metabolism (Clarke and Knowles 1980, Pugh and Knowles 1983, Frankenberg et al. 2002, Baureder et al. 2012). Regulation of the *katA* gene is under control of the HypR and Spx transcriptional activators, particularly in H_2_O_2_ stressed cells, but is independent of heme availability (Verneuil et al. 2005, Kajfasz et al. 2012, Baureder et al. 2014). It has also been shown that the catalase polypeptide (KatA) accumulates in *E. faecalis* during exponential phase independent of heme, but heme-supplemented cultures contain almost double the amount of catalase as nonsupplemented cultures. During stationary growth phase, the KatA polypeptide is degraded in the absence of heme while it remains stable in heme-rich cultures (Frankenberg et al. 2002, Baureder et al. 2014). Taken together, these observations indicate that heme is not only essential for activity but also stabilizes the catalase protein.

The *E. faecalis* catalase is a homotetrameric protein with each subunit binding one molecule of heme and a proline residue (P28) has been indicated as being essential to its activity (Hakansson et al. 2004, Baureder and Hederstedt 2012, Baureder et al. 2014). While the structure of catalase in *E. faecalis* has been solved, there is still a limited understanding regarding heme trafficking or how catalase assembly occurs *in vivo* (Brugna et al. 2010, Baureder et al. 2014). A transposon mutant library was used to screen for potential genes involved in catalase biogenesis including heme chaperone(s) and transporter(s) (Baureder and Hederstedt 2012). Five loci appear to indirectly impact catalase activity in *E. faecalis*, including *rnjA* and *srmB*, both of which are involved in RNA turnover (Condon 2010, Roux et al. 2011), *npr* which encodes for an NADH peroxidase important for endogenous H_2_O_2_ detoxification (La Carbona et al. 2007, Wasselin et al. 2022), the stress and virulence regulator *etaR* (Teng et al. 2002), and the oligopeptide transporter *oppBCDF*, which has been proposed to moonlight as a low affinity heme transporter (Letoffe et al. 2006, Baureder and Hederstedt 2012). Future investigations into the OppBCDF transporter capacity to import heme, alone or in conjunction with CydCD, and their roles in virulence should be investigated further. Other than *katA*, no single gene was essential for catalase function as all mutants retained some level of activity, suggesting that heme importers and chaperones were either missed in the screen or are encoded by multiple functionally redundant genes (Baureder et al. 2014).

While most strains of *E. faecalis* encode a heme-dependent catalase (catalase_h_), a manganese-dependent catalase has been identified and partially characterized in *E. faecium* (catalase _Mn_). However, the catalase_Mn_ does not appear to protect *E. faecium* from either endogenous or exogenous H_2_O_2_ stress (Wasselin et al. 2022). We used the BV-BRC database to search for homologues of *E. faecalis* OG1RF catalase_h_ (OG1RF_RS06790) and *E. faecium* DO catalase_Mn_ (HMPREF0351_10525) in other enterococci. Of the 25 species, only three species other than *E. faecalis* were found to encode a catalase_h_, albeit with varying degrees of prevalence. Specifically, catalase_h_ was found in 26% of *E. casseliflavus*, 3% of *E. gallinarum*, and 83% of *E. saccharolyticus* strains, and in no *E. faecium* strains, confirming previous results (Baureder and Hederstedt 2013) (Fig. 2). Interestingly, several of the species of enterococci that did not encode catalase_h_ had a high degree of prevalence for catalase_Mn_, including *E. faecium* (74%), *E. avium* (92%), *E. durans* (98%), and *E. gallinarum* (92%) (Fig. 2). A small percentage (7%) of *E. faecalis* strains also encoded a catalase_Mn_ and 97% of *E. casseliflavus* encoded catalase_Mn_, indicating that some strains of *E. faecalis* and *E. casseliflavus* possess both types of catalases. In *Vagococci*, 1% of the sequenced strains encoded catalase_h_ and no strains were found to encode catalase_Mn_, in *Tetragenococci*, 8% encode catalase_h_ and 33% encoded a catalase_Mn_ homologue sharing 40%–50% amino acid identity with *E. faecium* catalase_Mn_, and no *Melissococcus* strains encode either type of catalase. Future studies should confirm catalase activity and determine the mechanism of detoxification of catalase_Mn_, investigate the evolutionary significance of catalase_h_ and catalase _Mn_ in different enterococcal species, and determine possible differences in the contribution to bacterial fitness of catalase_h_ and catalase _Mn_ in different environments the enterococci inhabit.

## Heme toxicity is managed by an export system and heme sensing regulator

Despite heme being a nutrient cofactor, intracellular heme levels must be tightly controlled, as heme can be toxic to cells at high concentrations. In *E. faecalis* OG1RF, heme toxicity is managed by an ABC-type heme efflux transporter HrtAB (OG1RF_RS02770-RS02775) and heme-sensing TetR family regulator FhtR (OG1RF_RS02765) (Saillant et al. 2021). FhtR (*E. faecalis* heme transport regulator) exerts negative control over *hrtAB* in a heme-dependent manner; when intracellular heme levels rise, heme-bound FhtR is released from the *hrtAB* promoter region and transcription is activated (Saillant et al. 2021). The heme exporter is comprised of two subunits referred to as HrtA and HrtB for heme regulated transport. HrtA is the ATPase and HrtB is an FtsX-like permease. Expression of *hrtAB* is higher in the presence of heme, hemoglobin, and blood as well as within the gastrointestinal tract of mice suggesting that heme efflux might be important to *E. faecalis* during systemic infections and gut colonization. HrtAB is a member of the MacB family of efflux pumps in which instead of exporting heme directly from the cytoplasm, HrtB binds to heme trapped in the membrane and HrtA uses ATP to change confirmation of the heme bound complex to release heme into the extracellular environment (Nakamura et al. 2022). Due to the lipophilic nature of heme, it is possible heme may be able to passively diffuse across the cell membrane (Donegan et al. 2019). Similar to *E. faecalis, Lactococcus lactis* expresses an HrtAB heme exporter and no high affinity heme import system has been described (Joubert et al. 2014). Several studies have thus suggested that in some Gram-positive bacteria, there is not a need for an active heme uptake system, but that heme can become membrane associated, diffuse across the membrane, or be removed via an HrtAB type exporter (Joubert et al. 2014, Saillant et al. 2021, Nakamura et al. 2022). In conjunction with the recent discovery that CydCD binds and transports heme from the membrane, it is worth considering that in *E. faecalis*, heme is not scavenged from the environment via high affinity heme transporters, but rather heme becomes associated with the membrane, diffuses across, is transported via CydCD, or is exported by HrtAB (Joubert et al. 2014, Saillant et al. 2021, Nakamura et al. 2022, Wu et al. 2023).

Using BV-BRC and the same criteria as before, we found no other enterococci encoding FhtR. In a previous study, the *E. faecalis* FhtR was found to be more closely related to orthologues found in Vagococci (we found four species with >59% amino acid identity) and *Carnobacterium* sp than to potential orthologues in *E. faecium* (we found strains with <40% amino acid identity), *E. hirae* (we found strains with <40% amino acid identity), or *E. avium* (we found strains with <40% amino acid identity) (Saillant et al. 2021). In contrast to FhtR, we identified orthologues of HrtA (>50% amino acid identity) and an associated permease with lower similarity to HrtB (40%–50% amino acid identity) throughout several species of enterococci (Fig. 2). Using the *E. faecium* DO reference genome, we found that the most similar ATPase (58.6% amino acid identity) and permease (48% amino acid identity) components to HrtAB were indeed genetically linked to each other and to a TetR-type regulator (HMPREF0351_11396–11398) sharing 14.7% amino acid identity with the *E. faecalis* FhtR. It is important to note that both *E. faecalis* and *E. faecium* can tolerate high levels of heme in upwards of 50 µg/ml (∼74 µM heme) (Okorochenkov et al. 2013). Thus, it is possible that *E. faecium*, and possibly other enterococci, possess interconnected heme efflux/heme-sensing systems orthologous or analogous to HrtAB/FhtR.

## Heme may be an important source of iron for *E. faecalis* in systemic infections

Considering that heme is the most abundant form of iron in mammalian hosts, it is unsurprising that some of the most successful pathogenic bacteria employ multiple and diverse strategies to obtain heme from the environment to use as an iron source (Runyen-Janecky 2013, Sheldon and Heinrichs 2015, Choby and Skaar 2016, Hare 2017, Chatterjee et al. 2020, Jochim et al. 2020, Flannagan et al. 2022). Recently, our group showed that *E. faecalis* encodes at least five iron uptake systems that, collectively, are important for host colonization but that iron starvation in a strain lacking all five iron transporters can still be overcome by heme supplementation (Brunson et al. 2023). While the mechanisms of heme acquisition in *E. faecalis* remain elusive, as discussed above, results from this investigation leave little doubt that heme is an important source of iron to *E. faecalis* during infection.

Before heme can be used as an iron source, the iron atom must first be liberated from the porphyrin ring, which in bacteria is typically accomplished by a family of enzymes known as bacterial heme oxygenases (Lyles and Eichenbaum 2018). However, *E. faecalis* does not encode any of the characterized canonical or noncanonical heme oxygenases. On the other hand, all *E. faecalis* genomes encode a potential orthologue (OG1RF_RS05575) of a recently described family of enzymes identified in Gram-negative bacteria, known as anaerobilin synthase, that utilize radical *S*-adenosylmethionine methyltransferase to open the porphyrin ring and free the iron ion (LaMattina et al. 2016, Mathew et al. 2019, 2022, Brimberry et al. 2021). The best-characterized enzyme of the family, ChuW, was first described in *E. coli* and shares ~44% amino acid identity with *E. faecalis* RS05575. While the *E. faecalis* ChuW orthologue is often annotated as a coproporphyrinogen synthase (anaerobic heme synthesis) or as a heme chaperone, we suspect RS05575 functions as a heme-degrading enzyme under anaerobic conditions. Our lab is currently investigating the potential functions of RS05575, including heme degradation and chaperone activities. Moreover, we found that all species of *Enterococcus, Vagococcus, Melissococcus*, and *Tetragenococcus* encode an orthologue of RS05575 with varying degrees of prevalence (40%–100%), which suggests that heme degradation or other functions of this enzyme are advantageous to all enterococci and closely related genera that reside in the guts of animals.

In addition to this putative anaerobilin synthase, some enterococcal isolates encode *hemH*, a ferrochelatase whose exact function in the enterococci is unknown. In some bacteria, ferrochelatases are involved in heme biosynthesis (Choby and Skaar 2016) while in anaerobes such as *Porphyromonas gingivalis*, ferrochelatase (encoded by *ihtB*) was shown to degrade extracellular heme to liberate the iron atom (Dashper et al. 2000). We speculate that like the *P. gingivalis* IhtB, the enterococcal HemH homologue works in the reverse reaction and does not synthesize heme but rather degrades it due to the absence of other genes in the heme biosynthetic pathway in their genomes. In a study using comparative genomics to identify bacterial fitness factors in gut, urinary tract, and blood isolates, *hemH* was found in isolates from all three sites, but enriched in urinary tract isolates compared to gut and blood isolates (Sharon et al. 2023). The mechanistic function and role of *E. faecalis* HemH in virulence in different host niches should be investigated to gain a better understanding of when or if this auxiliary protein enhances virulence or interspecies competition. We used HemH (RS09495) from *E. faecalis* V583 to search for potential homologues in other enterococci and found that HemH is present in 62% of *E. faecalis* strains, 96% of *E. cecorum*, 100% of *E. dongliensis*, and *E. hulanensis*, and 52% of the Vagococci (Fig. 2). Yet, several other enterococci, including *E. faecium*, do not encode HemH. This uneven distribution among the enterococci begs the question of what role does HemH play in enterococcal niche adaptation and why is it absent in some species but highly prevalent in others?

## Other hemoproteins of enterococci

Despite the significance of heme as a cofactor and iron source to bacteria, the heme-binding proteome is not well-defined. This includes canonical heme proteins that bind heme with a high affinity and require heme for activity or that transport heme, referred to as heme proteins, and those that transiently bind heme or bind heme with low affinity, but heme is not essential for activity, referred to as heme-binding proteins. The development of click chemistry in which proteins are specifically labeled using biorthogonal chemical reactions combined with the power of large-scale proteomics has recently helped expand the number of known heme proteins and heme-binding proteins in bacteria (Parker and Pratt 2020, Wilkinson et al. 2023). This method was used to label heme with different probes to identify heme-binding proteins in *E. faecalis* V583 with five previously characterized proteins, namely HrtA, HrtB, CydA, CydC, and CydD, confirmed to have the capacity to bind heme (Wilkinson et al. 2023). Additionally, several new potential heme proteins and heme-binding proteins were identified, including the putative anaerobilin synthase OG1RF_RS05575 mentioned above, the peroxiredoxin AhpC that protects cells from organic peroxide and H_2_O_2_ stressors, and the type I glyceraldehyde-3-phosphate dehydrogenase (GAPDH). While heme was not anticipated to interact with AhpC (La Carbona et al. 2007)—the solved crystal structure of *E. faecalis* AhpC provides no indication of a heme-binding motif (Pan et al. 2018)—GAPDH has been shown to bind heme in mammalian cell lines (Chakravarti et al. 2010) and purified GAPDH from the lactic acid bacteria *Streptococcus gordonii* and *Streptococcus suis* was shown to bind heme *in vitro* (Hannibal et al. 2012, Slezak et al. 2020). Furthermore, four proteins with no previous association with heme were also pulled down using heme-based probes including the copper chaperone CopZ, an *N*-acetylmuramoyl-l-alanine amidase, a d-ala-d-ala carboxypeptidase, and a transcriptional regulator from the UvrC family. Potential heme-binding motifs within each of these proteins was determined using the HeMoQuest web server but these predictions still need to be experimentally validated (Paul George et al. 2020, Wilkinson et al. 2023). In the future, this powerful system can be refined to expand the list of heme-binding proteins in enterococci by assaying under different environmental conditions. This should include under aerobic versus anaerobic conditions, extreme iron starvation, planktonic versus biofilm lifestyles, and in assessing cell wall associated and secreted proteins.

## *Enterococcus faecalis* polymicrobial and host–pathogen interactions are influenced by heme

Because enterococci are natural residents of the complex gut microbiota and are frequently isolated from extra intestinal sites in polymicrobial infections, it is important to also consider the role heme and hemoproteins play in cross-bacterial and host–pathogen interactions. On this note, two recent studies have shed new light into the importance of heme as a metabolite in *E. faecalis* interspecies and host interactions. In the first study, the Kline lab showed that *E. faecalis* produces significantly more biofilm when grown in mixed cultures with *S. aureus* (Ch’ng et al. 2022). This phenomenon was shown to be associated with the presence of both *S. aureus* and *E. faecalis* factors. Specifically, *S. aureus* hemoproteins were degraded by secreted *E. faecalis* gelatinase, which resulted in heme-mediated activation of aerobic respiration by cytochrome *bd*. The authors concluded that aerobic respiration enhanced biofilm formation by increasing the ATP supply necessary for the synthesis of energy costly biofilm matrix and adhesion proteins such as EpaOX and EbpABC. Furthermore, secreted gelatinase was shown to break down hemoglobin, providing *E. faecalis* with a heme source of host origin (Ch’ng et al. 2022).

In the other study, the Zackular lab showed that coinfection of the mouse gut with *Clostridioides difficile* enables *E. faecalis* to access both heme and oxygen in the otherwise anoxic and low heme gut environment, resulting in robust interspecies biofilms (Khalili et al. 2017, Smith et al. 2024). This interspecies cooperation is accomplished by *C. difficile* production of toxins that damage the gut epithelium leading to an influx of hemoglobin/heme and diffusion of oxygen into the gut. As a result, *E. faecalis* utilizes heme and oxygen for aerobic respiration, which leads to increased biofilm formation and overall fitness gain, a phenotype that is reliant upon *E. faecalis* CydAB (Smith et al. 2024). In turn, *E. faecalis* assists *C. difficile* by metabolizing intestinal arginine (via the arginine deiminase system) and secreting ornithine, which promotes *C. difficile* colonization and increased toxin production (Smith et al. 2022). This synergistic relationship results in increased severity of infection and poor outcomes in patients with *C. difficile* colitis who are also heavily colonized with *E. faecalis*, particularly VRE strains (Smith et al. 2022, 2024). Yet, an *E. faecalis* transposon mutant lacking *cydA* (*cydA*::Tn) displayed no fitness defect in a mouse single species gut colonization model, indicating that under normal circumstances aerobic respiration is not a fitness factor for *E. faecalis* within the gut (Smith et al. 2024).

Notably, two separate studies have uncovered that *E. faecalis* engages in extracellular electron transfer (EET) as a method for energy generation, distinct from heme and cytochrome activation (Keogh et al. 2018, Pankratova et al. 2018, Hederstedt et al. 2020). Moreover, it has been shown that heme negatively impacts ferric reductase activity of *E. faecalis* EET, a phenomenon that is relieved in a cytochrome-deficient strain (Hederstedt 2022). While *E. faecalis* EET has yet to be demonstrated *in vivo*, a recent study with *Listeria monocytogenes* showed that an EET-deficient mutant displayed a competitive fitness defect in the mouse gastrointestinal tract (Light et al. 2018). Thus, it is possible that *E. faecalis* can also utilize EET to generate energy within the gut, provided heme and oxygen are in low enough levels to prevent cytochrome activation.

In contrast to the studies discussed above, another group found that growing *E. faecalis* in the presence of heme led to sensitization to the oxidative burst of neutrophils (Painter et al. 2017), indicating that aerobic respiration may be detrimental to *E. faecalis* at sites with high neutrophil recruitment. However, this study was performed with planktonic cultures and failed to consider if cells within a biofilm can be protected from these effects. Therefore, it is possible that at infection sites where *E. faecalis* persists in a polymicrobial biofilm lifestyle, such as the wound bed or on the surface of a urinary catheter, heme acquisition, and aerobic respiration may enhance the virulence of *E. faecalis* like what was observed in cocolonization with *C. difficile* in the gut.

Heme is both necessary and cytotoxic to the mammalian host due to its ability to promote and propagate the formation of dangerous ROS, particularly the capacity of heme to cause lipid peroxidation of eukaryotic membranes (Kumar and Bandyopadhyay 2005). Thus, just as free iron and other metals are tightly regulated by the host at cellular and systemic levels to avoid toxicity, so is heme. In mammals, free hemoglobin is quickly bound by haptoglobin, a protein that traffics hemoglobin to hepatocytes and macrophages for degradation (Andersen et al. 2017, Mozzi et al. 2018) while any free heme is sequestered by hemopexin and trafficked to the liver for catabolism (Morgan 1976, Tolosano et al. 2010). Expression of both haptoglobin and hemopexin are induced by IL-22 in response to inflammation during infection to restrict the availability of heme as a nutrient and iron source to bacterial pathogens (Datta 2017, Sakamoto et al. 2017, Mozzi et al. 2018). As of now, how enterococci compete for access to heme during infection remains largely unknown for several reasons, many of which have been discussed above. First, the major drivers of heme acquisition in enterococci are still poorly understood. Second, while the degradation of host and bacterial hemoproteins by *E. faecalis* gelatinase was shown to be important for heme acquisition, this phenomenon was not observed in the majority of strains evaluated, including strains positive for gelatinase. Moreover, at least two strains that lacked gelatinase activity were found to retain the ability to use hemoglobin to enhance biofilms (Ch’ng et al. 2022). Notably, we found that 84% of *E. faecalis* strains in the BV-BRC database encode *gelE* (OG1RF_07835) but no other species of enterococci as well as Vagococci, Melissococci, or Tetragenococci can produce gelatinase (Fig. 2). All together this is highly suggestive that while gelatinase might play a significant role in liberating heme from hemoproteins in *E. faecalis*, it is clearly not the sole mechanism. We propose there must be alternative ways that *E. faecalis* can access heme from hemoproteins. One possible mechanism is through the production of H_2_O_2_, which oxidizes hemoglobin to methemoglobin causing the release of heme from the protein, a process that has been demonstrated in *Streptococcus pneumoniae* (McDevitt et al. 2020). Furthermore, extended incubation of heme with H_2_O_2_-producing *S. pneumoniae* revealed that this process can eventually lead to heme degradation (Alibayov et al. 2022, Womack et al. 2024). Of note, *E. faecalis* generates low levels of H_2_O_2_ that is enhanced when grown on alternative sugars such as glycerol and galactose and during severely dysregulated metabolism, likely due to superoxide dismutase conversion of hydroxyl radicals to H_2_O_2_ (La Carbona et al. 2007, Colomer-Winter et al. 2017). Thus, it is conceivable that *E. faecalis* may also rely on net production of H_2_O_2_ to release and subsequently degrade heme from hemoproteins.

## Hemolysins of enterococci

The points discussed above indicate that *E. faecalis* can passively utilize free heme or liberate heme from hemoproteins, but little is really understood regarding additional approaches that may be taken by *E. faecalis* or other enterococci to increase bioavailable heme, such as the production of hemolysins. Lysis of red blood cells by hemolysins increases a wealth of nutrients available to enterococci, including heme. In general, the *Enterococcus* genus is categorized as gamma hemolytic, indicative of weak or no hemolysis, although enterococcal strains displaying either alpha or beta hemolysis have been isolated from clinical studies (Semedo et al. 2003, Creti et al. 2004, Gok et al. 2020, Hashem et al. 2021, Zarzecka et al. 2022). In fact, plasmids or chromosomal pathogenicity-associated islands have been shown to possess noncore genes, known as cytolysins, associated with hemolysis and/or killing of other Gram-positive bacteria (Shankar et al. 2004). Cytolysin genes include two operons that are genetically linked but transcribed in opposite orientation. The regulatory genes *cylR1* and *cylR2* are transcribed in the leftward direction and the structural genes *cylL(L), cylL(S), cylM, cylB, cylA*, and *cylI* are transcribed in the rightward direction. The *cylL(L)* and *cylL(S)* genes encode immature peptides that form the active cytolytic toxin. The *cylM* product post-translationally modifies the immature peptides prior to secretion, and the *cylB* gene product is localized to the membrane and processes the polypeptide. The *cylA* gene codes for another secreted protein that cleaves the peptides to form the mature toxin and *cylI* encodes an immunity protein that protects the parent cell from the toxin (Shankar et al. 2004, Van Tyne et al. 2013). Across several clinical studies, the association of hemolytic *E. faecalis* isolated at different infection sites has demonstrated a higher prevalence of cytolysin positive *E. faecalis* from nonsystemic infections such as wound or urinary tract infections than from systemic infections such as bacteremia or infective endocarditis (Huycke and Gilmore 1995, Archimbaud et al. 2002, Creti et al. 2004). Conversely, one such study demonstrated an enrichment of hemolytic enterococci from the gut of hospitalized patients compared to enterococci isolated from healthy controls in the community (Creti et al. 2004). Taken together this may indicate that while the hospital environment selects for hemolytic enterococci in the gut and in some localized infections of patients, this same activity may be detrimental to enterococci in systemic infections.

Most clinical studies that investigated genotype/phenotype association of hemolytic enterococci used the presence of *cylA* as an indicator of hemolysis/cytolysis activity. While the presence of *cylA* gene is often associated with beta hemolysis, some *cylA* positive strains are not hemolytic and strains that are *cylA* negative can exhibit beta hemolysis (Chai et al. 2019, Iseppi et al. 2020). We used CylL(L) (RS02570) and CylL(S) (RS02575) of *E. faecalis* V583 to determine the prevalence of these peptides. Both CylL(L) and CylL(S) were found in about 27% of *E. faecalis* strains but in no other enterococci (Fig. 2). Apart from cytolysin genes, the core genome of *E. faecalis* harbors three genes annotated as or with significant similarity to hemolysins, (OG1RF_RS07205, OG1RF_RS02335, and OG1RF_RS03705) but none of these gene products have been characterized. OG1RF_RS07205 encodes a putative hemolysin III (HlyIII) sharing ∼69% amino acid similarity with hemolysin III of *Bacillus cereus*, which was shown to oligomerize and create pores at the surface of red blood cells (Baida and Kuzmin 1996). OG1RF_RS02335 is annotated as *hlyC*, a putative transporter involved in the export of hemolysins and other molecules. OG1RF_RS03705 belongs to the TlyA family rRNA (cytidine-2`-O)-methyltransferase and is annotated as hemolysin A with homologues found in *B. cereus, Streptococcus agalactiae*, and *Streptococcus sanguinis*. Genes coding for HlyIII, HlyC, and HlyA are found at high prevalence in all 25 species of enterococci used in our searches (75% or more strains within a given species) and can be also found in nearly all Vagococci and Tetragenococci.

## Concluding remarks

The purpose of this review was to provide a comprehensive overview of the current understanding of the significance of heme in enterococci. Equally important was to identify existing knowledge gaps in the field and offer a roadmap for future investigations. Because *E. faecalis* is by far the most studied species of the genus, we searched for *E. faecalis* hemoproteins in other enterococci to have a better perspective of the diversity and importance of heme within the genus. We found that several of these hemoproteins, including the well-known catalase_h_ and CydABCD are nearly ubiquitous to *E. faecalis* but completely absent or part of the accessory genome of other enterococci. We suspect that the uneven distribution of known and suspected hemoproteins among the enterococci hints at crucial differences in their evolutionary history and the different selective pressures within environments encountered by ancestral strains. A notable observation that arose from these comparisons was the absence of cytochromes and heme-dependent catalase in *E. faecium* (Baureder and Hederstedt 2013) considering that both *E. faecalis* and *E. faecium* reside in the human gut and are capable of causing multiple opportunistic infections. Despite *E. faecium* exhibiting a higher prevalence for vancomycin/multidrug resistance, *E. faecalis* is more abundant in the gut and far more prevalent in human infections (Arias and Murray 2012, Huycke 2014). Furthermore, *E. faecalis* has demonstrated greater virulence in animal models compared to *E. faecium* (Garsin et al. 2014, Fiore et al. 2019). Future studies could investigate whether these differences are, at least in part, due to *E. faecalis’* enhanced ability to exploit host heme to its advantage.

## Supplementary Material

xtae019_Supplemental_Files
