# Supplementary material for: Heme utilization by the enterococci
Source: FEMS Microbes. 2024 Jul 2;5:xtae019. doi: 10.1093/femsmc/xtae019 (PMC11282960; doi:10.1093/femsmc/xtae019)
Supplement: xtae019_Supplemental_Files [file xtae019_supplemental_files.zip › FEMSMC-2024-011.R2 one sentence summary.docx]

The significance of heme to Enterococcus faecalis is reviewed while also identifying the prevalence of hemoproteins throughout the enterococci and highlighting gaps in knowledge in enterococcal mechanisms of heme homeostasis.
