# Supplementary material for: Heme utilization by the enterococci
Source: FEMS Microbes. 2024 Jul 2;5:xtae019. doi: 10.1093/femsmc/xtae019 (PMC11282960; doi:10.1093/femsmc/xtae019)
Supplement: xtae019_Supplemental_Files [file xtae019_supplemental_files.zip › FEMSMC-2024-011.r2 supplementary data - reviews and author response.docx]

**Reviews of manuscript FEMSMC-2024-011**

**Reviewer 1**

**Recommendation: Minor Revision**

This review is on a topic of rather wide interest and timely because substantial knowledge and information have accumulated concerning heme metabolism in enterococci and the research field is active with a plethora of recent publications. The most recent reviews on heme and lactic acid bacteria, including enterococci, are about 10 years old. The present review is the first focused on enterococci and heme. The gem of the review is the presentation and critical discussion of very new findings. The content, text and figures are overall satisfactory, but there are a few flaws and room for improvement of the manuscript concerning specificity and correctness.

Specific comments, including suggestions for improvement of the manuscript.

1. Introduction. Several reviews on heme and lactic acid bacteria, comprising also the genus *Enterococcus*, were published in the period 2012-2013. This should be acknowledged in the Introduction and reference 1 should be accompanied in the reference list with the very relevant publications; Gruss et al (2012) Adv Microbial Physiology 61, 69-124 doi.org/10.1016/B978-0-12-394423-8.00003-2; Baureder & Hederstedt (2013) Adv Microbial Physiology 62, 1-43 doi.org/10.1016/B978-0-12-410515-7.00001-9.

2. Figure 1, legend, line 428. Change text to: Established and predicted systems of heme acquisition, utilization and export in enterococci. Line 435-436. Delete this sentence, since the text does not belong to the figure legend.

3. Figure 1. Heme Import: It is unclear if CydCD might mediate transport of heme. It is therefore misleading to indicate this function of CydCD in the figure. Energy Generation: Cytochrome bd is not a proton pump but generates a proton gradient. Therefore, remove the arrow indicating proton transport.

4. Figure 2. State how and on what basis the phylogenetic tree on the lefthand side was generated. Was 16S rRNA sequence data used?

5. Title and throughout the text. The used word enterococci is not a name and therefore should not have a capital letter, in contrast to the name of the genus *Enterococcus* (which shall be spelled with a capital letter and in italic font).

6. The abstract text. Line 28-31. Change text to:………tetrapyrrole ring ligating an iron ion and has important……….systems. Heme is a cofactor for several enzymes and can be a major iron source…… Lines 32-35. Change to:……. some species of the genus *Enterococcus* are opportunistic pathogens………due to their ability to……….for growth. Some enterococci encode ……………, which may contribute……..

7. Lines 44-45. Change to:……cofactor for certain aerobe ………generation, such as sensing….

8. Line 49. Change to:…….cells and many other proteins (8).

9. Line 51. Change to: …..distribution of heme makes mechanisms……

10. Line 54-56. Change to: The enterococci are gut dwelling Gram-positive…….years ago and have been co-evolving……….Currently, the genus *Enterococcus* comprises a little……..species and representatives are found in the gut of virtually…….

11. Line 66. Change to:…..enzymes that can aide…..

12. Line 72. Add reference 48 here.

13. Lines 83-86. The three heme groups of cytochrome bd are all positioned in CydA. See recent reviews on the topic and add at least one to the reference list. For example, Borisov et al (2021) Antioxidants& Redox Signaling 34, 1280-1318. Change text to:….cytochrome bd consists of two polypeptides, CydA and CydB, and the CydA contains the three heme groups of the enzyme. The oxidase activity of the enzyme generates a transmembrane protein gradient that drives………

14. Line 91. Is reference 21 appropriate 1for the statement?

15. Line 94. Change to:……genome sequences of 66……..

16. Lines 107-108. Change to:……encode CydA and CydB….

17. Line 118. It has been demonstrated also in *E. faecalis* that CydC is required for assembly of cytochrome bd. See and add here reference 33.

18. Lines 119-121. Delete the sentence. I.e., change to:….activity (27-32). A recent study……

19. Line 135. After the sentence, add one or more references to support the statement.

20. Line 143. Is reference 34 valid here? Use instead, for example, Baureder & Hederstedt (2013) Adv Microbial Physiology 62, 1-43 doi.org/10.1016/B978-0-12-410515-7.00001-9

21. Line 146. Check if references 35 and 36 are meaningful here.

22. Line 147-153. Change to:…….been shown that the catalase polypeptide (KatA) accumulates……..stationary growth phase the KatA polypeptide is degraded……….remains stable in heme-rich…….that heme stabilizes the catalase protein.

23. Lines 169 – 184. Provide the numbers for percentage without a decimal, because the data does not provide that accuracy.

24. Line 203-204. Change to:……and the same criteria as before, we found no other……

25. Line 216. For the statement, add reference 41.

26. Line 248. Change to:…..while in anaerobes such as……

27. Section starting on line 266. It is of importance in the review to discriminate between canonical heme proteins and heme-binding proteins. Canonical heme proteins bind heme in stochiometric amounts with very high affinity and require heme for function. This is in contrast to proteins that can bind heme with low affinity, perhaps transiently, and do not depend on heme for activity or that transport heme. Clarify this point in the text, perhaps in the first sentences of the section.

28. Line 274. Change to:….synthase OG1RF_RS05575…..

29. Line 279. Change to………from the lactic acid bacteria *Streptococcus*……

30. Lines 287-290. This text is difficult to read. Change for example to:…..environmental conditions. This should include aerobic vs. anaerobic conditions, extreme iron starvation, planktonic vs. biofilm lifestyle, and accessing cell-bound and secreted proteins.

31. Section starting on line 291. In 2017, it was shown (Pankratova et. al., Electrochemistry Commun 75, 56-59) that *E. faecalis* cells can perform extracellular electron transfer (EET). The following year this discovery was established by more detailed experiments and shown to be influenced by heme, since cytochrome bd activity attenuates the EET to electrodes and ferric ions (Pankratova et. al., Biochemistry 57, 4597-4603). See also Hederstedt (2023) Microorganisms 11, 106. It is highly relevant to bring up EET, because the process seems important for the role and function of *E. faecalis* in, for example, the gut. EET can drive syntrophic growth and metabolism and therefore is of significant medical concern in the context of heme and the microbiome, see the Discussion in Pankratova et. al., (2018) Biochemistry 57, 4597-4603. The aspect of EET would fit well into the discussion about host-microbe and microbe-microbe interactions (Fig. 1 and lines 291-402).

32. Line 333. Change to:…..hemoglobin in mammals binds to …..

33. Lines 354-355. Change to:….generates extracellular H_2_O_2_ that is enhanced when grown on, for example, glycerol and galactose (44).

34. Line 355. Is reference 80 relevant to use here?

35. The text under section “*E. faecalis* polymicrobial and host-pathogen interactions are influenced by heme” is very long. Perhaps, the story would be more easy to follow if the text is split into two sections. I.e., starting with a new section, with for example the name “Hemolysins”, on line 357.

36. Line 370-372. Change to:…..secretion and the gene cylB product in the membrane processes the polypeptide. The cylA…..

37. Line 282, Change to……in the gut and in some localized infections in patients, this same…

38. Lines 388-389. Change to:….(RS02575) of *E. faecalis* V583 to determine…..prevalence of these polypeptides. Both CylL(L) and CylL(S) were found…..

39. Lines 382-393. Change to:…..none of the gene products have been characterized.

40. Line 400. Change to:…….are found at high prevalence in all 25 species……

41. Line 404. Change to:……….of the review was to…….

42. Lines 414-415. Change to:…..the absence of cytochromes and heme-dependent catalase in…….

43. Line 415. It has been demonstrated before that *E. faecium* isolates lack the genes for cytochrome bd and catalase (Baureder & Hederstedt, Adv Microbial Physiology 62, 1-43 doi.org/10.1016/B978-0-12-410515-7.00001-9). It could be stated in the review that this finding is confirmed.

**Reviewer 2: Delphine Lechardeur, INRAE**

**Recommendation: Major Revision**

The review of D. Brunson and J. Lemos, « Heme utilization by the Enterococci » describes the different utilizations of the porphyrin heme in Enterococci. Heme is an essential cofactor of several enzymes and also a toxic molecule, which metabolism and homeostasis must be tightly regulated by several bacteria. Interestingly, while expressing hemoproteins, enterococci cannot synthezise heme. They have developped strategies to scavenge heme from their environments while controling its toxicity.

The review is well written, complete and containing interesting new informations about the prevalence of hemoproteins in Enterococci.

Some paragraphs should be corrected/rewritten to include recent data :

Line 118 and fig. 1 : The work of Wu et al. (doi: 10.1038/s41589-023-01314-5) shows, based on a crystallographic study, that CydDC is a highly specific heme transporter required for assembly and maturation of cytochrome bd.

Line 164 and Fig. 1. Indirect evidences established the possibility that heme could enter gram positive bacteria by diffusion without the need of specific importers (Saillant et al., doi: 10.1128/mBio.03392-20). This hypothesis should be discussed.

Line 191 and Fig. 1. The crystal of HrtBA has been obtained by Nakamura et al. (doi: 10.1073/pnas.2123385119) and shows that heme is not not transported across the membrane from the cytoplasm to the extracellular space but is captured in the membrane. HrtBA functions as a barrier to the accumulation of heme in the membrane and in the cytoplasm. This mechanism also suggest that heme can enter the bacteria without the need of dedicated transporters (see above).

**Editor Decision: Minor Revision**

**Author's Response to Decision Letter for (FEMSMC-2024-011)**

Comments to the Author

This review is on a topic of rather wide interest and timely because substantial knowledge and information have accumulated concerning heme metabolism in enterococci and the research field is active with a plethora of recent publications. The most recent reviews on heme and lactic acid bacteria, including enterococci, are about 10 years old. The present review is the first focused on enterococci and heme. The gem of the review is the presentation and critical discussion of very new findings. The content, text and figures are overall satisfactory, but there are a few flaws and room for improvement of the manuscript concerning specificity and correctness.

Specific comments, including suggestions for improvement of the manuscript.

1. Introduction. Several reviews on heme and lactic acid bacteria, comprising also the genus Enterococcus, were published in the period 2012-2013. This should be acknowledged in the Introduction and reference 1 should be accompanied in the reference list with the very relevant publications; Gruss et al (2012) Adv Microbial Physiology 61, 69-124 doi.org/10.1016/B978-0-12-394423-8.00003-2; Baureder & Hederstedt (2013) Adv Microbial Physiology 62, 1-43 doi.org/10.1016/B978-0-12-410515-7.00001-9. These references have been added to the Introduction as suggested (Line 54-57).

2. Figure 1, legend, line 428. Change text to: Established and predicted systems of heme acquisition, utilization and export in enterococci. Line 435-436. Delete this sentence, since the text does not belong to the figure legend. Modified as suggested (Line 462-469)

3. Figure 1. Heme Import: It is unclear if CydCD might mediate transport of heme. It is therefore misleading to indicate this function of CydCD in the figure. Energy Generation: Cytochrome bd is not a proton pump but generates a proton gradient. Therefore, remove the arrow indicating proton transport. We removed the arrow as suggested. Regarding the role of CydCD in heme transport, we believe there is sufficient evidence supporting its association with heme uptake. In fact, Reviewer 2 provides recent paper that shows CydCD does transport heme and we have modified the text and the figure to reflect this information.

4. Figure 2. State how and on what basis the phylogenetic tree on the lefthand side was generated. Was 16S rRNA sequence data used? The phylogenetic tree was generated using the BV-BRC bacterial genome tree tool. A representative genome from each species was chosen and 1000 genes were used in the Codon Tree method in which both amino acid and DNA sequences from single copy genes were analyzed using RAxML. We added this information to the figure legend.

5. Title and throughout the text. The used word enterococci is not a name and therefore should not have a capital letter, in contrast to the name of the genus Enterococcus (which shall be spelled with a capital letter and in italic font). Made the correction throughout.

6. The abstract text. Line 28-31. Change text to:………tetrapyrrole ring ligating an iron ion and has important……….systems. Heme is a cofactor for several enzymes and can be a major iron source…… Lines 32-35. Change to:……. some species of the genus Enterococcus are opportunistic pathogens………due to their ability to……….for growth. Some enterococci encode ……………, which may contribute…….. Modified for clarity. (Line 28-37).

7. Lines 44-45. Change to:……cofactor for certain aerobe Done ………generation, such as sensing…. Changed to ..important roles such as energy generation, sensing and defense… (Line 45-46).

8. Line 49. Change to:…….cells and many other proteins (8). Done as suggested. (Line 50)

9. Line 51. Change to: …..distribution of heme makes mechanisms…… Done as suggested. (Line 52)

10. Line 54-56. Change to: The enterococci are gut dwelling Gram-positive…….years ago and have been co-evolving……….Currently, the genus Enterococcus comprises a little……..species and representatives are found in the gut of virtually……. Modified as recommended. (Line 58-61).

11. Line 66. Change to:…..enzymes that can aide….. Done as suggested. (Line 70)

12. Line 72. Add reference 48 here. Done as suggested. (Line 76)

13. Lines 83-86. The three heme groups of cytochrome bd are all positioned in CydA. See recent reviews on the topic and add at least one to the reference list. For example, Borisov et al (2021) Antioxidants& Redox Signaling 34, 1280-1318. Change text to:….cytochrome bd consists of two polypeptides, CydA and CydB, and the CydA contains the three heme groups of the enzyme. The oxidase activity of the enzyme generates a transmembrane protein gradient that drives……… We added a reference and made all modifications as suggested. (Line 87-96)

14. Line 91. Is reference 21 appropriate 1for the statement? This reference is appropriate.

15. Line 94. Change to:……genome sequences of 66…….. Done as suggested. (Line 101)

16. Lines 107-108. Change to:……encode CydA and CydB….Done as suggested. (Line 114-115)

17. Line 118. It has been demonstrated also in E. faecalis that CydC is required for assembly of cytochrome bd. See and add here reference 33. Done as suggested. (Line 130)

18. Lines 119-121. Delete the sentence. I.e., change to:….activity (27-32). A recent study…… This section has been vastly revised based on comments from both reviewers. (Line 127-142).

19. Line 135. After the sentence, add one or more references to support the statement. Done as suggested. (Line 145)

20. Line 143. Is reference 34 valid here? Use instead, for example, Baureder & Hederstedt (2013) Adv Microbial Physiology 62, 1-43 doi.org/10.1016/B978-0-12-410515-7.00001-9 Replaced reference as suggested. (Line 152).

21. Line 146. Check if references 35 and 36 are meaningful here. References have been removed. (Line 154-155)

22. Line 147-153. Change to:…….been shown that the catalase polypeptide (KatA) accumulates……..stationary growth phase the KatA polypeptide is degraded……….remains stable in heme-rich…….that heme stabilizes the catalase protein. Done as suggested. (Line 157)

23. Lines 169 – 184. Provide the numbers for percentage without a decimal, because the data does not provide that accuracy. Modified as suggested. (Changed throughout)

24. Line 203-204. Change to:……and the same criteria as before, we found no other……Done as suggested. (Line 225)

25. Line 216. For the statement, add reference 41. The suggested reference is for in vivo catalase maturation; we don’t think it is necessary (or correct) to support the statement.

26. Line 248. Change to:…..while in anaerobes such as……Done as suggested. (Line 269)

27. Section starting on line 266. It is of importance in the review to discriminate between canonical heme proteins and heme-binding proteins. Canonical heme proteins bind heme in stochiometric amounts with very high affinity and require heme for function. This is in contrast to proteins that can bind heme with low affinity, perhaps transiently, and do not depend on heme for activity or that transport heme. Clarify this point in the text, perhaps in the first sentences of the section. Modified as suggested (Line 287-290).

28. Line 274. Change to:….synthase OG1RF_RS05575….. Done as suggested. (Line 298)

29. Line 279. Change to………from the lactic acid bacteria Streptococcus…… Done as suggested. (Line 303)

30. Lines 287-290. This text is difficult to read. Change for example to:…..environmental conditions. This should include aerobic vs. anaerobic conditions, extreme iron starvation, planktonic vs. biofilm lifestyle, and accessing cell-bound and secreted proteins. Modified as suggested. (Line 311-312)

31. Section starting on line 291. In 2017, it was shown (Pankratova et. al., Electrochemistry Commun 75, 56-59) that E. faecalis cells can perform extracellular electron transfer (EET). The following year this discovery was established by more detailed experiments and shown to be influenced by heme, since cytochrome bd activity attenuates the EET to electrodes and ferric ions (Pankratova et. al., Biochemistry 57, 4597-4603). See also Hederstedt (2023) Microorganisms 11, 106. It is highly relevant to bring up EET, because the process seems important for the role and function of E. faecalis in, for example, the gut. EET can drive syntrophic growth and metabolism and therefore is of significant medical concern in the context of heme and the microbiome, see the Discussion in Pankratova et. al., (2018) Biochemistry 57, 4597-4603. The aspect of EET would fit well into the discussion about host-microbe and microbe-microbe interactions (Fig. 1 and lines 291-402). We incorporated new text discussing EET as suggested (Line 342-350).

32. Line 333. Change to:…..hemoglobin in mammals binds to ….. Text has been modified for clarity (Line 363-364)

33. Lines 354-355. Change to:….generates extracellular H2O2 that is enhanced when grown on, for example, glycerol and galactose (44). Text has been edited for greater clarity. (Line 385-388)

34. Line 355. Is reference 80 relevant to use here? We believe so. This paper shows that E. faecalis does produce low levels of intracellular H202 when grown in glucose, and that metabolic dysregulation enhances H202 production. The text has been edited for greater clarity. (Line 388)

35. The text under section “E. faecalis polymicrobial and host-pathogen interactions are influenced by heme” is very long. Perhaps, the story would be more easy to follow if the text is split into two sections. I.e., starting with a new section, with for example the name “Hemolysins”, on line 357. Text was separated as suggested. (Line 390)

36. Line 370-372. Change to:…..secretion and the gene cylB product in the membrane processes the polypeptide. The cylA….. Done as suggested. (Line 404-405)

37. Line 282, Change to……in the gut and in some localized infections in patients, this same… Done as suggested. (Line 414-415)

38. Lines 388-389. Change to:….(RS02575) of E. faecalis V583 to determine…..prevalence of these polypeptides. Both CylL(L) and CylL(S) were found….. Done as suggested. (Line 421-422)

39. Lines 382-393. Change to:…..none of the gene products have been characterized. Done as suggested. (Line 425-426)

40. Line 400. Change to:…….are found at high prevalence in all 25 species…… Done as suggested. (Line 433)

41. Line 404. Change to:……….of the review was to……. Done as suggested.

42. Lines 414-415. Change to:…..the absence of cytochromes and heme-dependent catalase in……. Done as suggested. (Line 448-449)

43. Line 415. It has been demonstrated before that E. faecium isolates lack the genes for cytochrome bd and catalase (Baureder & Hederstedt, Adv Microbial Physiology 62, 1-43 doi.org/10.1016/B978-0-12-410515-7.00001-9). It could be stated in the review that this finding is confirmed. We have now cited this reference (Line 449) as suggested and included a line in the text in the catalase section (line 186).

Reviewer: 2

Comments to the Author

The review of D. Brunson and J. Lemos, « Heme utilization by the Enterococci » describes the different utilizations of the porphyrin heme in Enterococci. Heme is an essential cofactor of several enzymes and also a toxic molecule, which metabolism and homeostasis must be tightly regulated by several bacteria. Interestingly, while expressing hemoproteins, enterococci cannot synthezise heme. They have developed strategies to scavenge heme from their environments while controlling its toxicity.

The review is well written, complete and containing interesting new informations about the prevalence of hemoproteins in Enterococci.

Some paragraphs should be corrected/rewritten to include recent data :

Line 118 and fig. 1 : The work of Wu et al. (doi: 10.1038/s41589-023-01314-5) shows, based on a crystallographic study, that CydDC is a highly specific heme transporter required for assembly and maturation of cytochrome bd. https://www.nature.com/articles/s41589-023-01314-5#Sec2 New citation and corresponding text added as suggested. (Line 137-142)

Line 164 and Fig. 1. Indirect evidences established the possibility that heme could enter gram positive bacteria by diffusion without the need of specific importers (Saillant et al., doi: 10.1128/mBio.03392-20). This hypothesis should be discussed. 191 and Fig. 1. The crystal of HrtBA has been obtained by Nakamura et al. (doi: 10.1073/pnas.2123385119) and shows that heme is not transported across the membrane from the cytoplasm to the extracellular space but is captured in the membrane. HrtBA functions as a barrier to the accumulation of heme in the membrane and in the cytoplasm. This mechanism also suggest that heme can enter the bacteria without the need of dedicated transporters (see above). Text discussing structure, mechanism, and the possibility of heme diffusion across the membrane have been added as suggested (Lines 212-225).

**Reviews of manuscript FEMSMC-2024-011.r1**

**Reviewer 1**

**Recommendation: Minor Revision**

The revised manuscript meets to a satisfactory level the comments from the two reviewers. During the read of the revised manuscript I found text that need minor correction.

Line 29. Change the sentence to:….oxygen binding molecule…….

Line 81. CydB is not a cytochrome. Therefore, change the headline to: Energy Generation by Cytochrome bd.

Line 82. Replace the word produce with conserve.

Line 87. The sentence is confusing and must be amended. The enzyme activity of cytochrome bd results in a translocation of protons, i.e., contributes to the creation of a transmembrane electrochemical gradient that can drive ATPase activity. As written, the transmembrane gradient drives release of two protons. This is not the case!

Line 89. Change the sentence to:…..it does not contain heme, it is ……

Line 93. Cytochrome bd is not essential for generation of the gradient. The gradient can form also in the absence of cytochrome bd activity. Correct the statement.

Line 110. Change sentence to:…..prevalence of CydA…..

Line 129. Change to: …mutants disrupted in either…..

Line 204. Delete the word fully.

Figure 2, legend, lines 475 and 481. The text appears here to some extent be redundant.

Line 308. enterococci (no capital letter)

References. Data is missing in several instances. For example, page numbers for references 93, 94 and 95.

**Reviewer 2: Delphine Lechardeur, INRAE**

**Recommendation: Accept**

The authors made the requested changes to their review.

**Editor Decision: Minor Revision**

**Author's Response to Decision Letter for (FEMSMC-2024-011.r1)**

Comments to the Author

The revised manuscript meets to a satisfactory level the comments from the two reviewers. During the read of the revised manuscript I found text that need minor correction.

Line 29. Change the sentence to:….oxygen binding molecule……. The change has been made.

Line 81. CydB is not a cytochrome. Therefore, change the headline to: Energy Generation by Cytochrome bd. The change has been made.

Line 82. Replace the word produce with conserve. The change has been made.

Line 87. The sentence is confusing and must be amended. The enzyme activity of cytochrome bd results in a translocation of protons, i.e., contributes to the creation of a transmembrane electrochemical gradient that can drive ATPase activity. As written, the transmembrane gradient drives release of two protons. This is not the case! The change has been made.

Line 89. Change the sentence to:…..it does not contain heme, it is …… The change has been made.

Line 93. Cytochrome bd is not essential for generation of the gradient. The gradient can form also in the absence of cytochrome bd activity. Correct the statement. The sentence has been removed.

Line 110. Change sentence to:…..prevalence of CydA….. The change has been made.

Line 129. Change to: …mutants disrupted in either….. The change has been made.

Line 204. Delete the word fully. The change has been made.

Figure 2, legend, lines 475 and 481. The text appears here to some extent be redundant. The text has been edited.

Line 308. enterococci (no capital letter) The change has been made.

References. Data is missing in several instances. For example, page numbers for references 93, 94 and 95. References have been fixed.

Reviewer: 2

Comments to the Author

The authors made the requested changes to their review.
